# Dynamic Morphology of Dilated Ascending Aorta and its Implications for Proximal Landing During Thoracic Endovascular Aortic Repair

**DOI:** 10.1177/15266028241292462

**Published:** 2024-11-13

**Authors:** Denis Skrypnik, Moritz S. Bischoff, Katrin Meisenbacher, Matthias Hagedorn, Samuel Kilian, Fabian Rengier, Florian Andre, Dittmar Böckler, Henning Steen

**Affiliations:** 1 Department of Vascular and Endovascular Surgery, University Hospital Heidelberg, Heidelberg, Germany; 2 Institute of Medical Biometry, Heidelberg University, Heidelberg, Germany; 3 Clinic for Diagnostic and Interventional Radiology, University Hospital Heidelberg, Heidelberg, Germany; 4 Clinic for Cardiology, Angiology and Pneumology, University Hospital Heidelberg, Germany

**Keywords:** thoracic endovascular aortic repair, TEVAR of ascending aorta, landing zone morphology, endografting of ascending aorta, dynamic morphology of ascending aorta

## Abstract

**Introduction:**

To improve the outcomes of thoracic endovascular aortic repair (TEVAR), we investigated the dynamic morphology of dilated and nondilated ascending aortas (AAs) to determine whether an appropriate proximal landing zone for TEVAR exists if the middle AA is dilated.

**Materials and Methods:**

Patients with dilated (diameter 40–50 mm) and nondilated (<40 mm) AAs underwent electrocardiogram-gated computed tomography angiography of the entire AA in the systolic and diastolic phases. For each plane of each AA segment, the maximal and minimal diameters in systole and diastole were recorded.

**Results:**

A total of 105 patients were enrolled (54% male; median age: 80 years [IQR 78–85]). A total of 35 patients were included in the dilated AA group (DG), and 70 patients were included in the nondilated AA group (n-DG). The aortic planes of the AA segment at the sinotubular junction (STJ) showed a more oval-shaped morphology compared with the distal planes of the same segment (the differences between the maximum and minimum diameters were 8.9% to 9.4% and 4.8% to 5.6%, respectively). If the mid-ascending aorta was dilated, the aortic segment at the STJ showed a more pronounced reversed-funnel 3D morphology, with a 14% (IQR 11%–19%) difference in diameters between the proximal and distal segmental planes shown in the n-DG and 18% (IQR 16%–22%, p<0.001) in the DG.

**Conclusion:**

If the middle AA is dilated, it is considered unsuitable to perform TEVAR using conventional endografts without additional proximal fixation in the aortic segment at the STJ due to its pronounced reversed-funnel segmental morphology. By contrast, the aortic segment at the brachiocephalic trunk seems to be promising for performing TEVAR using an endograft of the appropriate size and conformability. Moreover, endograft sizing using the average aortic diameter instead of the maximal AA diameter in an oval-shaped aortic plane morphology should be considered.

**Clinical Impact:**

If the middle ascending aorta is dilated, it is considered unsuitable to perform TEVAR with a proximal landing in the aortic segment at the sinotubular junction due to its pronounced reversed-funnel segmental morphology and high risk of type Ia endoleak and endograft migration. By contrast, the aortic segment at the brachiocephalic trunk seems to be promising for performing TEVAR due to its moderate funnel morphology using an endograft of appropriate size and conformability.

## Introduction

Even though open surgery remains the gold standard for the treatment of ascending aortic (AA) pathology, thoracic endovascular aortic repair (TEVAR) is a promising option in patients with a high risk profile for open surgery and an appropriate aortic morphology.^1,2^ Recently published studies have reported promising TEVAR outcomes with landings in the AA if a proximal nonpathological aortic segment of the AA is present.^3,4^ However, successful TEVAR outcomes are severely limited due to AA aneurysms or proximal landings in dilated AAs.^5^

Obtaining the 2- and 3-dimensional (2D and 3D) morphologies of the proximal landing zone (PLZ) in a nondilated AA is challenging due to the reversed-funnel shape of the segment at the sinotubular junction (STJ) and the slightly funnel-shaped segmental form of the AA at the brachiocephalic trunk (BT).^6^ It remains unknown whether a similar PLZ morphology can be secured if the middle AA is dilated, although small AA aneurysms with diameters of 40 to 50 mm have shown slow growth during long-term follow-up, and these could potentially be appropriate sites for proximal landing by means of TEVAR.^7^

Thus, the aim of this exploratory study was to investigate the dynamic morphology of dilated and nondilated AAs to determine whether an appropriate PLZ for TEVAR at the STJ and BT exists if the middle AA is dilated.

## Methods

### Study Design

We performed a retrospective, single-center analysis of prospectively collected clinical and computed tomography (CT)-based imaging data. To analyze aortic morphology over the whole cardiac cycle, we selected patients on whom electrocardiogram-gated computed tomographic angiography examinations (ECG-CTA) of the systole and diastole were performed due to critical stenosis of the aortic valve while planning for transcatheter aortic valve implantation. Our retrospective analyses of the prospectively cumulated data were approved by the local ethics committee (S-620/2018). We followed the Strengthening the Reporting of Observational Studies in Epidemiology (STROBE) standards for data presentation and reporting of study outcomes.

### Inclusion Criteria

This study included patients who were admitted for transcatheter aortic valve implantation planning between 1 July 2019 and 30 December 2020.

All patients underwent ECG-CTA of the entire AA and were divided into either a dilated (40–50 mm diameter) group (DG) or a nondilated (<40 mm diameter) group (n-DG).

### Exclusion Criteria

Patients with annuloaortic ectasia, connective tissue disease, a left ventricular ejection fraction of less than 30%, or calcinosis of more than 30% of the circumference of the AA were excluded. Moreover, patients were excluded if they had undergone previous heart surgery or had incomplete imaging of the AA. The patients with nondilated AAs who were analyzed in a previously published study were excluded from this study.

### Image Acquisition

ECG-gated CT angiography examinations of the heart and the ascending aorta were acquired using a 64-slice CT scanner (Philips IQon; Philips, Best, The Netherlands) when patients were in a supine position during inspiration breath hold. The protocol for the scanning parameters and contrast media was as follows: a tube potential of 120 kVp, automated tube current modulation, and 80 mL of iodinated contrast medium followed by a 50 mL saline bolus. Image reconstruction was performed at 5% steps of the RR interval with a slice thickness of 0.67 mm, a slice increment of 0.33 mm, and the IMR 1 Cardiac Routine kernel. The systole was assessed at 40% of the RR interval, while the diastole was assessed at 75% of the RR interval.

### Segmentation and Image Analysis

**Figure 1.**
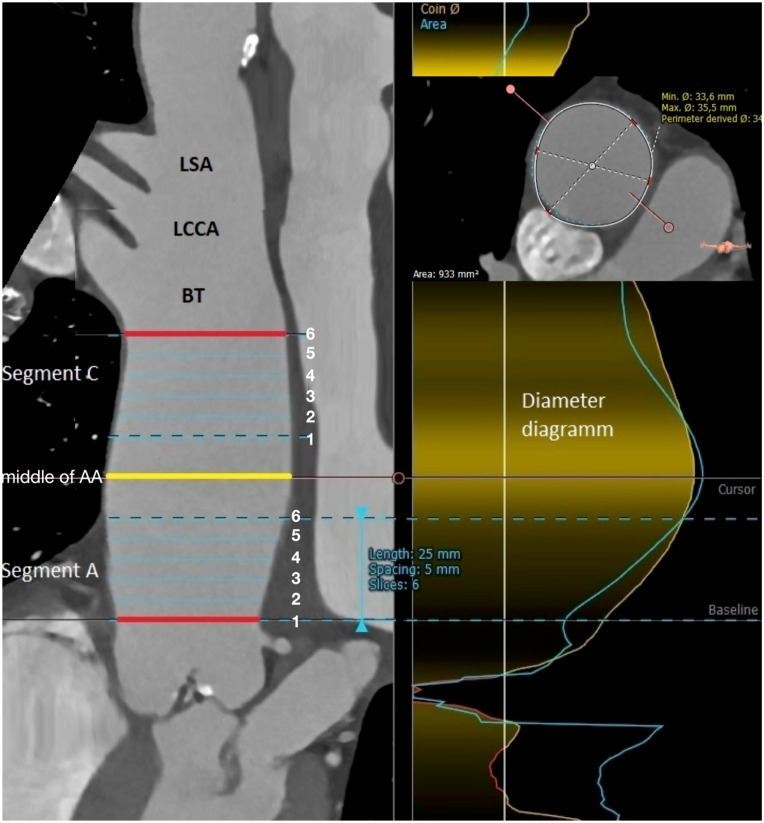
Segmentation of ascending aorta. Yellow line—aortic plane in the middle of ascending aorta. Bottom red line—aortic plane at sinotubular junction (proximal plane of the segment A). Upper red line—aortic plane at brachicephalic trunk (distal plane of the segment C). Blue lines—intermediate aortic planes. LSA, left subclavian artery; LCCA, left common carotid artery; BT, brachiocephalic trunk. Right figure panel—aortic diameter diagram.

The ECG-CTA of the entire AA in the systolic and diastolic phases was analyzed using 3mensio Vascular postprocessing software (Pie Medical Imaging BV, Maastricht, Netherlands). The image series was uploaded from the institutional database to a separate workstation, and 3D centerline reconstruction of the entire AA was performed. The AA segmentation was manually performed using the recommended 2.5 cm centerline length of each AA segment perpendicular to the centerline.^1,6^ The proximal plane of the most proximal AA segment (segment A) was at the STJ, and the distal plane of the distal AA segment (segment C) was at the edge of the BT (Figure 1). In all cases, the length of the AA and its maximal diameter were recorded. The maximal diameter was determined semiautomatically using a diameter diagram (Figure 1). To analyze the aortic plane dimensions within the aortic segments, the intermediate planes within a 2.5 cm segment were centered automatically with a 5 mm indention from each other and from the proximal and distal segmental planes (Figure 1).

For each plane of each segment, the maximum (D max) and minimum (D min) diameters and the segmental area during systole and diastole were recorded. Two independent study collaborators analyzed all image series. The section on “Statistical Analysis” describes how agreement between the collaborators was reached.

### Definitions

The length of the AA was defined as the centerline length between the STJ and BT. The aortic plane was defined as an automatically configured aortic slice positioned perpendicular to the centerline. An aortic segment was defined as a 3D part of the AA between the proximal and distal segmental planes with a centerline-based length of 2.5 cm^1^. The shapes of the aortic segments were determined as the difference between the dimensions of the distal and proximal aortic planes in systole and diastole. The aortic planes were defined as circular if all diameters were the same. If the diameters were different, the plane morphology was considered oval-shaped.

The length of the AA at its maximal dilatation was defined as the distance between the STJ and aortic plane that showed the maximal AA diameter. To measure how the proximal segmental plane was larger/smaller than the distal one, the relative difference between the proximal and distal segmental plane size values was calculated for segment A as (Dmax systolic plane 6—Dmax systolic plane 1)/Dmax systolic plane 6 and for segment C as (Dmax systolic plane 1—Dmax systolic plane 6)/Dmax systolic plane 1. These calculations consider the fact that the distal segmental plane is larger than the proximal one in segment A and the distal segmental plane of segment C is smaller than the proximal one.^6^

### Statistical Analysis

All analyses were performed using R version 4.2.0.^8^ All available patients with a dilated AA and those with a nondilated AA were matched at a 1:2 ratio. The matching was performed without replacements and considering the covariates of age, sex, and body surface area after DuBois.^9^ Optimal pair matching with the Mahalanobis distance was performed using the MatchIt package in R, which calls functions from the optmatch package.^10,11^ The quality of the matching procedure was assessed based on a graphical comparison of the covariate distribution within the 2 groups (dilated vs nondilated).

Two independent collaborators analyzed the image series of the included patients and extracted the data. Agreement between the 2 collaborators was graphically investigated by constructing Bland–Altman plots and by calculating intraclass correlation coefficients (ICCs). If the disagreement was striking, the extracted values were reassessed. The data set for the final analysis consisted of the mean values calculated from the collaborators’ measurements.

The descriptive measures comprised the median, first, and third quartiles (Q1 and Q3) for continuous variables, and the relative and absolute frequencies for categorical variables. Differences between the 2 groups regarding baseline variables were described using descriptive p-values. The Mann–Whitney U test was used for continuous variables and the Chi-square test was used for categorical variables.

The descriptive p-values for the differences between 2 variables within 1 group (eg, between the maximal systolic and maximal diastolic diameters shown in Supp. Table 4) were calculated according to the Wilcoxon signed-rank test for the null hypothesis that the median difference would equal zero. Nonparametric confidence intervals for the median of differences were calculated via bootstrapping with 1000 replicates using the R package boot.^12,13^

## Results

This study included 105 patients. After matching based on a 1:2 ratio, 35 patients were included in the dilated AA group (DG) and 70 were included in the nondilated AA group (n-DG). The majority of patients were male (54%, 57/105) with a median age of 80 years (IQR 78–85) and a median body surface area of 1.8 (IQR 1.7–2). Arterial hypertension (97%, 102/105), coronary heart disease (93%, 98/105), and cholesterolemia (70%, 74/105) were the most common cardiovascular risk factors. All demographic data are presented in Table 1. Arterial hypertension and hypercholesterolemia were more prevalent in the n-DG (100% vs 91%, p=0.01 and 77% vs 57%, p=0.03, respectively; Table 1). However, in the DG, there were more stroke patients (23% vs 4%, p=0.04), and this group showed a greater AA length compared with the n-DG (79 vs 67 mm, p<0.001). Moreover, the DG showed a larger AA diameter (43 vs 36 mm, p<0.001). The length of the AA at maximal dilatation in this group was 48% of the entire AA length, that is, almost in the middle of the AA (Table 1).

**Table 1.** Patient Demographic Data and Morphological Settings.^a^

Variable	Nondilatation group (N=70)	Dilatation group (N=35)	Total (N=105)	p-value
	Median (Q1, Q3); % (n/N)			
Age, years	80.0 (78.0, 85.0)	79.0 (77.5, 84.5)	80.0 (78, 85)	0.52
Male	54% (38/70)	54% (19/35)	54% (57/105)	>0.99
Female	46% (32/70)	46% (16/35)	46% (48/105)	
BSA, m^2^	1.8 (1.7, 2.0)	1.9 (1.7, 2.1)	1.8 (1.7, 2.0)	0.90
BMI, kg/m^2^	25.3 (22.7, 29.4)	26.0 (24.0, 28.8)	26 (23, 29)	0.49
Hypertension	100% (70/70)	91% (32/35)	97% (102/105)	0.01
Coronary heart disease	91% (64/70)	97% (34/35)	93% (98/105)	0.26
PTCA	33% (23/70)	31% (11/35)	32% (34/105)	0.88
AF	43% (30/70)	43% (15/35)	43% (45/105)	>0.99
Previous stroke/TIA	9% (6/70)	23% (8/35)	13% (14/105)	0.04
COPD	10% (7/69)	20% (7/35)	13% (14/105)	0.16
Diabetes mellitus	23% (16/70)	26% (9/35)	24% (25/105)	0.74
Dialysis	1% (1/70)	3% (1/35)	2% (2/105)	0.61
Chronic renal insufficiency (Creatinine>1.2 mg/dL)	20% (14/70)	17% (6/35)	19% (20/105)	0.72
History of smoking	29% (20/70)	23% (8/35)	27% (28/105)	0.53
Hyperlipoproteinemia	77% (54/70)	57% (20/35)	70% (74/105)	0.03
Length of AA, mm	67.0 (61.0, 71.0)	79.0 (70.0, 85.0)	69.0 (63.0, 76.0)	<0.001
Maximal diameter of AA, mm	36.0 (33.8, 37.8)	43.1 (41.8, 45.5)	38 (35, 42)	<0.001
Zone of AA^b^ maximal diameter	n.a.	0.48 (0.42, 0.51)	n.a.	n.a.

Abbreviations: BSA, body surface area; BMI, body mass index; PTCA, percutaneous transluminal coronary angioplasty; AF, atrial fibrillation; TIA, transient ischemic attack; COPD, chronic obstructive pulmonary disease; AA, ascending aorta.

a Categorical data are presented as absolute numbers and percentage; continuous data are presented as median and first and third quartiles.

b Corresponds to the centerline-length of the ascending aorta from sinotubular junction to the maximal diameter plane in the dilatation-group.

### 2D Dynamic Morphology of Aortic Segments

The aortic dimension values showed considerable variability during the heart cycle, with larger AA dimensions at all segmental planes in the DG compared with the n-DG (Supp. Table 2). In both groups, the smallest systolic and diastolic dimensions were found at the STJ (proximal plane of aortic segment A) and the largest were located closer to the middle AA (Supp. Table 2).

**Figure 2.**
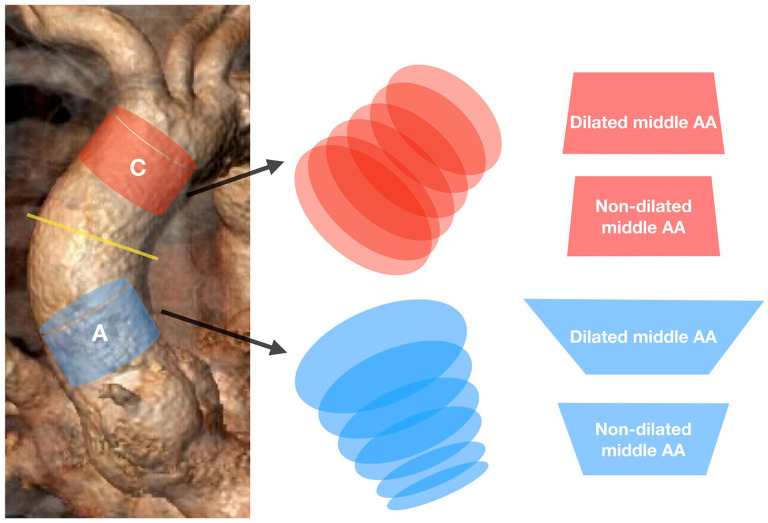
Graphical presentation of 2D and 3D aortic segmental morphology. Left panel—3D reconstruction of the ascending aorta. A (blue)—aortic segment at sinotubular junction. C (red)—aortic segment at brachiocephalic trunk. Yellow line—middle of the ascending aorta. Middle panel—graphical presentation of the 2D morphology of aortic planes of the aortic segments. Right panel—graphical presentation of the 3D morphology of aortic segments.

All segmental planes in both groups showed a 2D oval-shaped morphology during the heart cycle, with a median relative difference between the maximum and minimum diameters in different segmental planes of about 4.7% to 9.4% (Supp. Table 3). Meanwhile, the proximal planes of segment A showed a more pronounced oval-shaped morphology in the DG and n-DG, with median differences between the maximum and minimum diameters of 8.9% to 9.4% compared with the distal planes of the same segment (4.8%–5.6%) (Figure 2, middle panel, Supp. Table 3). This phenomenon was not observed in segment C, where the difference between the maximum and minimum diameters was similar for all segmental planes in the DG and n-DG (medians 4.7%–6.1%) (Figure 2, middle panel).

An analysis of the aortic dimensions in all segmental planes in the DG and n-DG revealed higher systolic values for both AA segments (Supp. Table 4). The median difference between the maximal systolic and maximal diastolic diameters in different segmental planes was 0.1 to 0.7 mm, and it was randomly distributed in both groups at all segmental levels, irrespective of the distance to the STJ and the phase of the cardiac cycle. In general, the larger systolic dimensions in different segmental planes were more pronounced in the DG compared with the n-DG in segment A (medians of 0.4–0.6 mm vs 0.1–0.5 mm) as well as in segment C (medians of 0.3–0.7 mm vs 0.2–0.4 mm) (Supp. Table 4).

### 3D Dynamic Morphology of Aortic Segments

Segment A showed a reversed-funnel shape and segment C showed a funnel shape in the DG and n-DG, respectively (Table 2, Figure 2, right panel). However, the diameter difference between the proximal and distal segmental planes was greater in the DG compared with the n-DG. The median relative diameter difference between the proximal and distal segmental planes for segment A in the n-DG was 14% (IQR 11%–19%) versus 18% (IQR 16%–22%, p<0.001) in the DG (Figure 2, right panel). Compared with segment A, this median relative diameter difference at segment C was smaller (n-DG: 4.8% at Q1, 2.4% and 7.6% at Q3; DG: 7.8% at Q1, 5.9% and 12% at Q3; p<0.001) (Figure 2, right panel). Thus, if the mid-ascending aorta was dilated, the aortic segments at the STJ and BT showed more pronounced noncylindrical morphology, with the greatest difference in diameters shown between the proximal and distal segmental planes at segment A in the DG.

**Table 2.** Segmental Shape of Ascending Aorta During the Heart Cycle.^a^

Segment		D^b^ systolic max, mm	Area systolic, mm^2^	D diastolic max, mm	Area diastolic, mm^2^
A	Nondilatation group	5.20 (4.40; 5.90)	266.50 (248.50; 289.00)	5.05 (4.65; 5.50)	248.95 (230.00; 263.99)
	Dilatation group	7.60 (7.30; 8.89)	460.05 (413.00; 496.00)	7.50 (7.10; 7.90)	452.50 (418.00; 472.60)
C	Nondilatation group	–1.70 (–2.00; –1.35)^c^	–100.43 (–113.50; –72.15)	–1.90 (–2.20; –1.60)	–102.00 (–111.35; –86.90)
	Dilatation group	–3.30 (–4.20; –2.90)	–212.00 (–241.50; –168.00)	–3.30 (–4.40; –2.80)	–215.75 (–235.00; –159.90)

The table presents 3D morphology during the heart cycle, wherein AA segment at STJ showed a reversed funnel shape and segment at BT showed a funnel shape in the DG and n-DG groups.

a Difference between distal and proximal maximal plane diameter and area is presented in mm as median and 95% CI (N=105).

Proximal plane diameter and area of segment A were smaller than the distal segmental plane values (reversed funnel form) (p<0.001 for all comparisons).

Proximal plane diameter and area of segment C were larger than the distal segmental plane values (funnel form) (p<0.001 for all comparisons).

b D, diameter.

c Negative values showed that distal diameter and area of segment C are smaller than proximal one.

In all cases, the image quality was sufficient for reliable measurements (N=105). The ICC was >0.9 for all values, showing good reproducibility for all measurements and high similarity for all values.

## Discussion

This study showed that due to the moderate dilatation of the AA reversed-funnel-shaped segmental morphology at the STJ aggravates, the most proximal AA segment showed a more pronounced 3D reversed-funnel shape compared with nondilated AAs. Meanwhile, the difference in aorta dimensions between the proximal and distal segmental planes increased from 14% in the n-DG to 18% in the DG.

A previously published study reported a reversed-funnel-shaped AA segment at the STJ for nondilated AAs in systole and diastole.^6^ This PLZ morphology, in association with the pulsatility of the proximal AA and the irregular 3D deformation and displacement of the aortic root during the heart cycle, predisposed patients to suboptimal endograft alignment, type Ia endoleaks, and retrograde type A aortic dissection (RTAAD).^14–18^

This is consistent with clinical reports, which have shown that the worst outcomes of TEVAR occur with proximal landings at the STJ.^19,20^ According to the data from the current study, the results of using TEVAR with proximal landings at the STJ in dilated AAs using conventional endografts without additional fixation could be even worse than expected. Otherwise, in a dilated AA, the 3D morphology of the PLZ at the STJ is more unsuitable for optimal endograft alignment than in a nondilated AA, and therefore, fewer patients have received TEVAR due to the unsuitable morphology of the PLZ. A meta-analysis conducted by Muetterties et al^5^ reported that only 5% of TEVAR procedures were performed due to AA aneurysms.

By contrast, the segment at the BT showed a slightly funnel-like shape in nondilated AAs, which is more appropriate for proximal landing using conventional endografts, and therefore, more patients with this appropriate morphology have received TEVAR.^6^ This finding confirmed a meta-analysis conducted by Zhu et al,^4^ who reported that 50% of TEVAR procedures were due to aneurysms of the aortic arch and using a proximal landing site in the AA.

Dilatation of the mid-ascending aorta was associated with a more pronounced funnel-shaped segmental morphology at the BT wherein the size differences between the proximal and distal segmental planes slightly increased from 8% in the n-DG to 12% in the DG. If endografts can eliminate this mismatch without causing bird-beaking or gaps at the proximal endograft edge, the noncylindrical morphology of this aortic segment should be acceptable.^21,22^ Moreover, considering the slow growth rate of small AA aneurysms, proximal landings in the distal AA that are associated with mild mid-ascending dilatation may be associated with promising TEVAR outcomes in early- and mid-stage follow-up.^7^

The precise sizing of the AA for TEVAR is essential. Excessive oversizing is associated with RTAAD and an early-stage mortality rate of >30%.^17^ A clinical study by Liu et al^23^ reported a reduced RTAAD rate due to accurate endograft sizing using the average aortic diameter instead of the maximal AA diameter in an oval-shaped aortic plane morphology, where the larger plane diameter was more than 5% greater than the smaller one. A previously published morphometrical study examining nondilated AAs reported an oval 2D aortic plane morphology of the proximal and distal planes for all AA segments, with a difference of >5% between the smaller and larger diameters in systole and diastole.^6^ However, in this study, the aortic morphology within the segments was not examined.

The data from the current study showed an oval 2D aortic plane morphology within the aortic segments in both the n-DG and the DG, with a difference of **≥**5% between the smaller and larger plane diameters, thus confirming the previously reported results. Moreover, the current data showed that the proximal segmental planes of the AA at the STJ in the DG and n-DG had an almost 2-fold more pronounced oval-shaped 2D morphology compared with the distal segmental planes of the same AA segment (8.9%–9.4% and 4.8%–5.6%, respectively). Thus, we should consider not only the reversed-funnel 3D morphology of the whole AA segment at the STJ, but also the irregular 2D segmental plane morphology along this segment.

The segment at the BT showed an oval 2D plane morphology at the proximal and distal segmental planes, which is consistent with previously reported observations.^6^ However, based on the current data, this 2D morphology might also be present along the whole segmental length in the n-DG and DG. Moreover, compared with the AA segment at the STJ, the segment at the BT showed a relatively uniform distribution of differences between the smaller and larger plane diameters along the whole segment (4.7%–6.8% for both groups).

The data from the current study support the measurement of the AA using ECG-CTA, thereby confirming the larger systolic dimensions compared with the diastolic dimensions at all levels of the AA in both the n-DG and the DG, wherein the maximal systolic diameter of the AA reached 40 to 45 mm.^6,24,25^ Considering that aortic sizes in males are 7% higher than those in females and endograft oversizing of up to 20% occurs when performing TEVAR due to aneurysms of the AA, the appropriate dimensions of off-the-shelf devices should be reconsidered.^26–30^

### Limitations

The present study has several limitations. The DG was 2 times smaller than the n-DG due to the availability of patients, and so to decrease the bias of our analysis of aortic size values, matching was performed at a 1:2 ratio after adjusting for covariates of age, sex, and body surface area. This study included patients with advanced age and arteriosclerosis of the AA, which might have influenced aortic stiffness. All patients had significant aortic stenosis, which might have influenced the direction and velocity of the ejected stroke volume. However, the direct influence of severe aortic stenosis on the aortic distensibility of noncalcified AAs as a function of aortic diameter change and arterial pressure was not reported in cases in which stroke volume and cardiac output were rescued.^31,32^ Furthermore, evenly distributed AA stiffness may introduce bias with respect to the absolute aortic size values, and this is unlikely to result in changes in the aortic size ratios. Thus, the 3D morphology of the aortic segments will probably stay the same. The DG showed significantly longer AAs, which might introduce bias in terms of 3D segmental morphology. With a shorter AA length, the distal planes of segment A (at the STJ) and the proximal planes of segment C (at the BT) were located at the mid-ascending aorta, where dilatation was the most pronounced. Finally, this study did not investigate AA angulation, longitudinal motions, or lateral deviations, which might be relevant for a complete description of the dynamic 3D morphology of the AA.

## Conclusions

If the middle AA is dilated, it is considered unsuitable to perform TEVAR in the AA using conventional endografts without an additional proximal fixation in the aortic segment at the STJ due to its pronounced reversed-funnel segmental morphology. By contrast, the aortic segment at the BT seems to be promising for performing TEVAR using an endograft of appropriate size and conformability. Moreover, in an oval-shaped aortic plane morphology, endograft sizing using the average aortic diameter instead of the maximal AA diameter should be considered.

## Supplemental Material

sj-docx-1-jet-10.1177_15266028241292462 – Supplemental material for Dynamic Morphology of Dilated Ascending Aorta and its Implications for Proximal Landing During Thoracic Endovascular Aortic Repair Supplemental material, sj-docx-1-jet-10.1177_15266028241292462 for Dynamic Morphology of Dilated Ascending Aorta and its Implications for Proximal Landing During Thoracic Endovascular Aortic Repair by Denis Skrypnik, Moritz S. Bischoff, Katrin Meisenbacher, Matthias Hagedorn, Samuel Kilian, Fabian Rengier, Florian Andre, Dittmar Böckler and Henning Steen in Journal of Endovascular Therapy
