# Supplementary material for: Dynamic Morphology of Dilated Ascending Aorta and its Implications for Proximal Landing During Thoracic Endovascular Aortic Repair
Source: J Endovasc Ther. 2024 Nov 13;33(3):1286–94. doi: 10.1177/15266028241292462 (PMC13176811; doi:10.1177/15266028241292462)
Supplement: sj-docx-1-jet-10.1177_15266028241292462 – Supplemental material for Dynamic Morphology of Dilated Ascending Aorta and its Implications for Proximal Landing During Thoracic Endovascular Aortic Repair [file sj-docx-1-jet-10.1177_15266028241292462.docx]

**Supp. Table 2. Segmental area and systolic/diastolic diameter values^a^**

| Segment | | A | | | | | | C | | | | | |
| --- | --- | --- | --- | --- | --- | --- | --- | --- | --- | --- | --- | --- | --- |
| Plane number^b^ | | 1 | 2 | 3 | 4 | 5 | 6 | 1 | 2 | 3 | 4 | 5 | 6 |
| Systolic Dmin, mm | Non-dilatation group | 27.1 (25.0, 28.6) | 28.8 (26.8, 30.5) | 30.1 (28.5, 31.7) | 31.4 (29.7, 33.2) | 32.3 (30.1, 34.2) | 32.9 (30.6, 35.0) | 32.6 (31.1, 34.5) | 32.4 (30.3, 34.4) | 32.0 (30.0, 33.9) | 31.5 (29.8, 33.5) | 31.1 (29.6, 32.8) | 30.8 (29.1, 32.6) |
|  | Dilatation group | 31.2 (28.5, 33.5) | 33.6 (31.4, 35.1) | 35.1 (33.5, 36.6) | 37.0 (35.0, 37.9) | 38.7 (36.5, 40.7) | 39.9 (38.0, 41.7) | 38.9 (37.1, 40.7) | 37.7 (36.0, 40.0) | 37.5 (34.9, 39.0) | 36.7 (34.5, 38.4) | 36.2 (34.0, 37.6) | 35.5 (33.2, 37.7) |
| Systolic Dmax, mm | Non-dilatation group | 29.6 (27.8, 31.6) | 31.2 (29.0, 32.7) | 32.3 (30.4, 33.9) | 33.6 (31.3, 35.7) | 34.3 (32.4, 36.4) | 35.3 (33.1, 37.0) | 35.1 (33.3, 36.9) | 34.7 (32.8, 36.3) | 34.1 (32.2, 35.9) | 33.9 (31.8, 35.6) | 33.7 (31.7, 34.9) | 33.0 (31.4, 35.0) |
|  | Dilatation group | 33.9 (32.7, 36.3) | 36.0 (34.5, 38.2) | 38.0 (36.6, 39.9) | 39.1 (38.3, 41.7) | 40.9 (39.6, 43.0) | 42.1 (40.7, 44.2) | 41.2 (39.7, 43.0) | 40.2 (38.7, 42.3) | 39.7 (37.7, 41.0) | 38.8 (36.2, 40.0) | 38.4 (35.3, 39.8) | 37.2 (35.5, 39.8) |
| Area systolic, mm^2^ | Non-dilatation group | 634.2 (551.1, 719.5) | 707.3 (610.0, 780.9) | 772.5 (683.8, 845.8) | 827.5 (727.5, 911.5) | 873.5 (766.5, 958.7) | 916.5 (814.2, 1019.5) | 911.2 (821.0, 997.5) | 874.0 (784.0, 982.4) | 864.5 (752.0, 948.5) | 843.6 (732.2, 931.6) | 821.5 (727.2, 902.2) | 817.0 (722.0, 891.9) |
|  | Dilatation group | 817.0 (758.0, 950.0) | 936.5 (869.2, 1071.7) | 1053.0 (961.0, 1178.5) | 1138.0 (1055.0, 1274.5) | 1221.9 (1139.5, 1386.1) | 1326.0 (1225.3, 1437.0) | 1246.0 (1168.0, 1363.5) | 1208.0 (1102.0, 1296.5) | 1169.0 (1016.5, 1250.0) | 1122.0 (978.8, 1204.8) | 1066.0 (945.9, 1166.0) | 1038.0 (932.6, 1181.8) |
| Diastolic Dmin, mm | Non-dilatation group | 27.4 (24.8, 29.2) | 28.6 (26.6, 31.1) | 29.9 (28.0, 32.2) | 31.0 (29.1, 33.2) | 32.0 (30.2, 34.1) | 32.8 (30.6, 34.8) | 32.6 (30.6, 34.7) | 32.1 (30.3, 34.2) | 31.8 (30.1, 33.6) | 31.4 (29.3, 33.1) | 31.1 (29.1, 32.7) | 30.6 (28.7, 32.6) |
|  | Dilatation group | 30.5 (29.6, 33.5) | 32.5 (31.5, 35.4) | 35.0 (33.0, 37.4) | 36.5 (34.9, 39.0) | 37.8 (36.5, 40.1) | 39.4 (37.8, 41.3) | 38.7 (36.8, 40.5) | 38.2 (35.8, 39.7) | 37.2 (34.4, 38.8) | 36.5 (33.8, 38.2) | 35.7 (33.2, 37.6) | 35.3 (32.8, 37.0) |
| Diastolic Dmax, mm | Non-dilatation group | 29.4 (27.9, 31.2) | 30.6 (28.8, 32.7) | 32.0 (30.2, 34.1) | 33.5 (31.2, 35.2) | 34.4 (32.3, 36.2) | 34.9 (32.9, 36.8) | 34.8 (33.2, 36.1) | 34.5 (32.1, 35.8) | 34.0 (31.8, 35.4) | 33.5 (31.3, 34.9) | 32.8 (31.0, 34.8) | 33.0 (30.7, 34.2) |
|  | Dilatation group | 33.8 (32.2, 36.2) | 35.1 (33.9, 37.8) | 37.2 (36.2, 39.7) | 39.0 (37.8, 41.1) | 40.5 (38.7, 42.4) | 41.9 (40.0, 43.1) | 40.9 (39.4, 42.2) | 40.0 (39.0, 41.5) | 38.9 (37.8, 40.5) | 38.3 (36.5, 40.0) | 37.5 (35.2, 39.3) | 37.2 (34.6, 39.0) |
| Area diastolic, mm^2^ | Non-dilatation group | 637.0 (535.5, 713.9) | 689.4 (595.8, 796.4) | 762.5 (668.4, 867.8) | 811.9 (722.5, 924.2) | 860.5 (768.5, 959.2) | 905.2 (798.8, 999.1) | 897.0 (803.0, 977.8) | 877.5 (771.9, 968.5) | 855.5 (741.5, 935.7) | 831.0 (717.2, 898.8) | 803.5 (704.5, 890.7) | 788.5 (707.0, 868.1) |
|  | Dilatation group | 822.0 (760.0, 971.5) | 902.5 (845.2, 1069.5) | 1008.0 (946.5, 1165.6) | 1112.9 (1036.5, 1259.8) | 1194.0 (1115.8, 1325.5) | 1303.0 (1191.7, 1425.7) | 1225.2 (1146.5, 1354.0) | 1212.9 (1094.8, 1291.1) | 1134.0 (1037.2, 1239.1) | 1113.0 (984.0, 1201.7) | 1061.0 (942.0, 1146.5) | 1051.8 (881.2, 1128.7) |

**The table presents larger AA dimensions at all segmental planes in the DG group compared to the n-DG group with the smallest systolic and diastolic dimensions at the STJ and the largest dimensions at the middle AA.**

DG, dilatation group; n-DG, non-dilatation group; STJ, sinotubular junction; AA, ascending aorta

^a^Data is presented as median and first and third quartile (Q1, Q3) (N=105)

^b^Segment A plane number 1 at sinotubular junction; Segment C plane number 6 at brachiocephalic trunk

Dilatation group show larger dimensions of all aortic planes (p<0.001 for all comparisons)

**Supp. Table 3.** Segmental plane asymmetry of ascending aorta during the heart cycle^a^

| Segment A | | | | | | | | | | | | |
| --- | --- | --- | --- | --- | --- | --- | --- | --- | --- | --- | --- | --- |
| Plane number | 1 | | 2 | | 3 | | 4 | | 5 | | 6 | |
|  | Systole | Diastole | Systole | Diastole | Systole | Diastole | Systole | Diastole | Systole | Diastole | Systole | Diastole |
| Non-dilatation group | 9.4% (6.5%, 12.9%) | 7.9% (5.2%, 11.8%) | 7.2% (5.6%, 9.7%) | 7.0% (4.8%, 9.1%) | 6.9% (5.1%, 9.3%) | 7.1% (4.8%, 9.6%) | 6.7% (4.3%, 8.8%) | 6.3% (4.6%, 9.1%) | 6.4% (4.3%, 8.2%) | 6.1% (4.7%, 8.4%) | 6.1% (4.4%, 7.8%) | 5.6% (4.2%, 7.5%) |
| Dilatation group | 8.9% (5.7%, 11.0%) | 8.6% (4.8%, 11.2%) | 6.9% (4.4%, 11.3%) | 7.0% (3.0%, 9.2%) | 7.1% (4.7%, 10.2%) | 6.0% (3.7%, 9.2%) | 5.8% (4.1%, 10.3%) | 5.9% (3.6%, 8.6%) | 6.0% (3.7%, 9.3%) | 5.2% (4.1%, 7.3%) | 6.2% (4.2%, 7.8%) | 4.8% (3.1%, 6.6%) |
| Segment C | | | | | | | | | | | | |
| Plane number | 1 | | 2 | | 3 | | 4 | | 5 | | 6 | |
|  | Systole | Diastole | Systole | Diastole | Systole | Diastole | Systole | Diastole | Systole | Diastole | Systole | Diastole |
| Non-dilatation group | 5.7% (4.1%, 7.7%) | 5.1% (3.5%, 6.7%) | 6.3% (4.0%, 8.3%) | 5.0% (3.8%, 7.3%) | 6.4% (4.4%, 7.7%) | 5.3% (3.5%, 7.5%) | 6.3% (4.8%, 8.0%) | 5.8% (3.9%, 7.7%) | 6.1% (4.7%, 8.0%) | 5.3% (4.1%, 6.6%) | 6.8% (4.8%, 8.8%) | 6.1% (3.6%, 8.1%) |
| Dilatation group | 5.6% (4.4%, 7.5%) | 4.8% (2.6%, 7.7%) | 5.6% (4.3%, 7.5%) | 4.9% (3.2%, 8.2%) | 5.3% (3.3%, 6.7%) | 4.7% (3.5%, 7.7%) | 5.0% (3.8%, 7.0%) | 4.9% (3.4%, 7.5%) | 5.4% (4.0%, 7.0%) | 5.0% (4.1%, 6.7%) | 6.5% (5.1%, 8.0%) | 4.8% (4.0%, 7.2%) |

**The table presents 2D oval-shaped morphology during the heart cycle, with a median relative difference between the maximum and minimum diameters in different segmental planes of 4.7-9.4%.**

^a^ segmental plane asymmetry was calculated as (D max-D min)/D max in the systole and diastole; data is presented as median and first and third quartile (Q1, Q3) (N=105)

**Supp. Table 4.** Difference between maximal systolic and maximal diastolic diameter^a^

| Segment A | | | | | | | | | | | | |
| --- | --- | --- | --- | --- | --- | --- | --- | --- | --- | --- | --- | --- |
|  |  | p-value |  | p-value |  | p-value |  | p-value |  | p-value |  | p-value |
| Plane number | 1 |  | 2 |  | 3 |  | 4 |  | 5 |  | 6 |  |
| Non-dilatation group | 0.30 (0.00; 0.60) | 0.045 | 0.10 (-0.05; 0.55) | 0.042 | 0.30 (0.10; 0.40) | 0.026 | 0.20 (0.10; 0.40) | 0.031 | 0.30 (0.10; 0.43) | 0.034 | 0.47 (0.15; 0.55) | <0.001 |
| Dilatation group | 0.40 (0.20; 0.90) | 0.035 | 0.50 (0.35; 1.00) | 0.028 | 0.40 (0.30; 0.70) | 0.075 | 0.40 (0.00; 0.80) | 0.044 | 0.60 (0.30; 0.90) | 0.022 | 0.60 (0.20; 0.80) | 0.001 |
| Segment C | | | | | | | | | | | | |
| Plane number | 1 |  | 2 |  | 3 |  | 4 |  | 5 |  | 6 |  |
| Non-dilatation group | 0.20 (0.00; 0.40) | 0.052 | 0.30 (0.10; 0.45) | 0.001 | 0.30 (0.10; 0.45) | <0.001 | 0.30 (0.10; 0.50) | <0.001 | 0.40 (0.27; 0.65) | <0.001 | 0.43 (0.30; 0.70) | <0.001 |
| Dilatation group | 0.50 (0.20; 0.90) | 0.004 | 0.40 (0.10; 0.70) | 0.026 | 0.30 (0.10; 0.50) | 0.002 | 0.40 (0.00; 0.60) | 0.027 | 0.40 (0.20; 0.80) | 0.027 | 0.70 (0.40; 1.00) | <0.001 |

**The table presents larger systolic aortic values compared to diastolic aortic values at the STJ and BT in the DG and n-DG groups.**

DG, dilatation group; n-DG, non-dilatation group; STJ, sinotubular junction; AA, ascending aorta

^a^Difference between maximal systolic and maximal diastolic diameter is presented in mm as median and 95% CI (N=105)

^b^Segment A plane number 1 was at sinotubular junction; Segment C plane number 6 was at brachiocephalic trunk
